# Refractory Celiac Disease Type II: A Case Report and Literature Review

**DOI:** 10.3389/fmed.2020.564875

**Published:** 2020-12-03

**Authors:** Richa Chibbar, Jordan Nostedt, Dana Mihalicz, Jean Deschenes, Ross McLean, Levinus A. Dieleman

**Affiliations:** ^1^Division of Gastroenterology, Department of Medicine, University of Alberta, Edmonton, AB, Canada; ^2^Division of Gastro, Department of Medicine, Beth Israel Deaconess Medical Center, Boston, MA, United States; ^3^Division of General Surgery, Department of Surgery, University of Alberta, Edmonton, AB, Canada; ^4^Department of Laboratory Medicine and Pathology, Cross Cancer Institute, University of Alberta, Edmonton, AB, Canada; ^5^Department of Laboratory Medicine, Royal Alexandra Hospital, University of Alberta, Edmonton, AB, Canada

**Keywords:** refractory celiac disease, EATL, ulcerative jejunitis, sigmoid perforation, immunophenotyping

## Abstract

We present an unusual case of 68-year-old male, who presented with acute abdomen, ulcerative jejunitis with perforation, and 2 months later with perforation of the sigmoid colon. We will also discuss difficulties in the delay in diagnosis of refractory celiac disease (RCD), specifically the atypical presentation, multiple surgeries, the consecutive failure of distinct therapeutic options, and multiple complications that occurred within the 3 months since first presentation.

## Introduction

Celiac disease (CeD) is an immune-mediated enteropathy triggered by gluten ingestion in genetically susceptible individuals. In the western population, the reported prevalence is 1% (1). CeD is recognized by classical clinical symptoms, features of malabsorption, and positive serology (anti-gliadin and anti-transglutaminase antibodies). The diagnosis is further supported by iron deficiency and characteristic endoscopic and histologic features (modified Marsh classification). The current mainstay of therapy is a gluten-free diet (GFD). Failure to respond after 6 months of a strict GFD, or nonresponsive celiac disease, is typically due to accidental gluten ingestion. Alternative diagnoses must also be considered including Crohn's disease, superimposed infection, bacterial overgrowth, and pancreatic exocrine insufficiency (2). However, 1–2% of those with CeD will have refractory celiac disease (RCD), defined as persistent or recurrent malabsorption, villous atrophy despite a strict gluten free diet (GFD) for 6–12 months (3, 4). RCD is further classified as either type 1 (RCD I) or type 2 (RCD II), where RCD I has a normal population of intraepithelial lymphocytes (IELs), while RCD II has aberrant IELs, which are clonal T-cells. Overall, RCD II is associated with a difficult treatment course and poorer prognosis, ulcerative jejunitis and higher rates (80%) of progression to enteropathic-associated T-cell lymphoma (EATL) within 5 years (5).

In those patients with undiagnosed CeD, RCD (including RCD II or ulcerative jejunoileitis) may be the initial presentation. RCD II is a rare entity and an acute presentation is atypical and should be a consideration in acute intestinal obstruction with jejunal involvement.

## Case

A 68-year-old gentleman with hypothyroidism, history of pelvic fracture, bilateral indirect inguinal hernia repair, and remote laparotomy for small bowel obstruction presented with acute onset abdominal pain and imaging findings of free intra-abdominal air with a large amount of free fluid and stranding in the proximal jejunum. He underwent resection of 25 cm of small bowel and a side-to-side anastomosis for a spontaneous jejunal perforation and areas of ulceration and stricturing proximally. The histologic findings were interpreted as nonspecific and no etiology for jejunal perforation was identified; however, it was thought to be secondary to intestinal ischemia due to adhesions from a previous laparotomy. No obvious laboratory abnormalities were noted at that time. He recovered well post-operatively and was discharged within a week.

He re-presented 6 weeks later with 30 lb weight loss, diarrhea, profound hypoalbuminemia (albumin of 10 g/L), and gastrointestinal bleeding with an elevated INR of 9 (not anticoagulated) that was subsequently corrected and normalized. Gastroscopy showed scalloping of the duodenal mucosa with flattened villi, featureless stomach and lack of prominent folds, suggestive of CeD or an infiltrative process. Biopsies from the duodenum were consistent with Celiac disease modified Marsh classification IIIc, while sections from the stomach, ileum, and colon showed features of lymphocytic gastritis, lymphocytic ileitis, and lymphocytic colitis, respectively. The IgA-anti-transglutaminase (IgA anti-tTG) was borderline elevated at 8.1 (Normal <7 U/mL) and HLA testing was positive for HLA1^*^05/DQB1^*^02 (alleles linked to CeD). Based on the initial presentation of jejunal perforation with ulcerative jejunoileitis (surgical specimen), histologic features of CeD, marked hypoalbuminemia, and coagulation abnormalities a working diagnosis of refractory CeD was rendered. He was not on Olmesartan (xi).

He was treated with a GFD, peripheral and parenteral nutrition, as well as intravenous corticosteroids, without improvement. Repeat endoscopies continued to demonstrate endoscopic and histological features compatible with active CeD. Though the IELs were less prominent, it is unclear if this was due to a gluten-free diet, corticosteroids, or sampling error.

**Figure 1:**
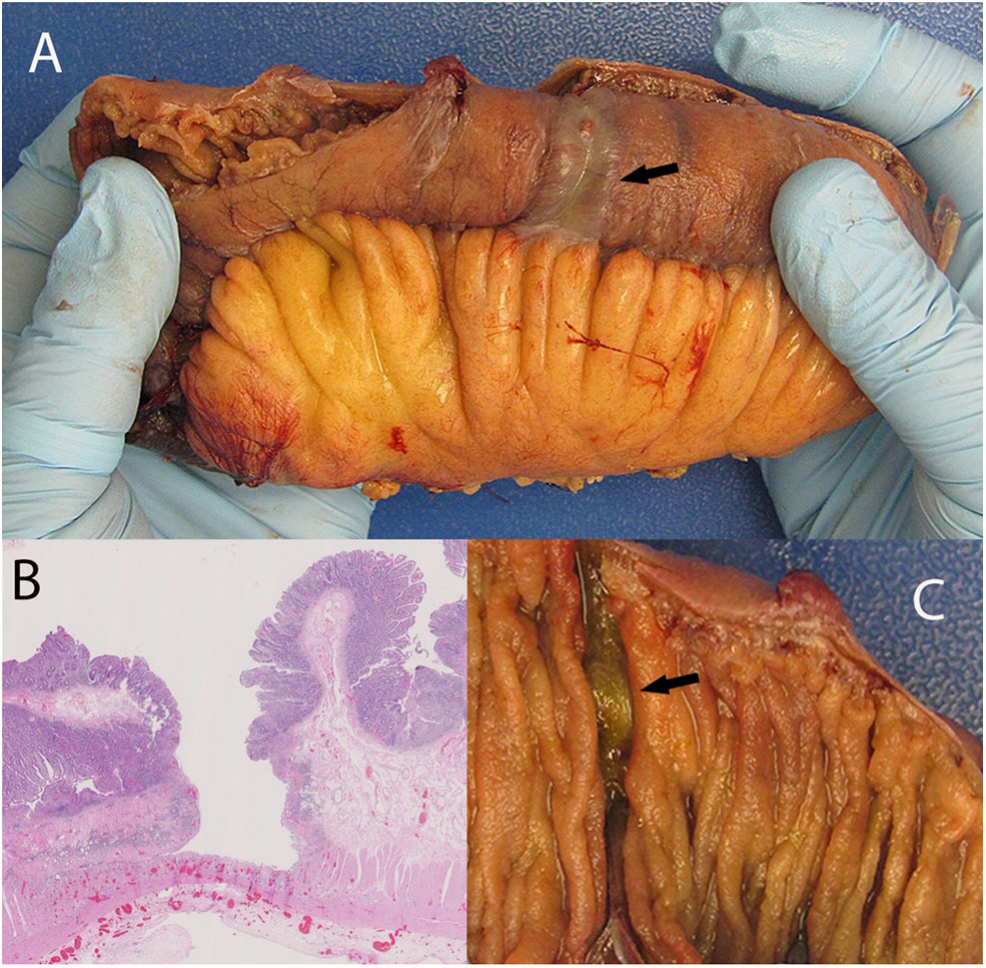
Discolored serosa mimicking localized ischemia (**A**; arrow) giving the appearance of annular circumferential rings. This is associated with a circumferential transverse ulcer (**C**, arrow). The discoloration is caused by congested vessels within remaining muscularis propria and adventitia situated under the ulcer base. **(B)** shows deep ulceration in the colon.

His admission was further complicated by occlusion of the IMA, confirmed on CT enterography. Colonoscopy to determine the source of rectal bleeding demonstrated probable ischemic colitis of the distal sigmoid colon. This procedure was complicated by sigmoid perforation resulting in exploratory laparotomy. In addition to lysis of adhesions, 15 cm of damaged small bowel was resected owing to intraoperative enterotomies, and 16.5 cm of sigmoid colon was resected for presumed ischemia. The small bowel also had an ischemic appearance, likely due to multiple circumferential serosal cyanotic stripes. Gross examination of the small intestinal mucosa showed circumferential transverse ulcerations. These were deep resulting in a very thin residual intestinal wall which coincided with the cyanotic stripes on the serosal aspect as observed by the surgeon. Microscopic examination showed extensive atypical IELs adjacent to the ulceration in otherwise grossly normal small intestinal mucosa. The sigmoid colon also showed ulceration with focal atypical IELs in grossly normal colon, as well as lymphocytic colitis. A repeat operation for abdominal washout revealed more annular ischemic appearing areas situated a few to several centimeters apart throughout the jejunum giving a striped appearance. There were as many as 15 discolored annular foci. The bowel wall appeared very thin and membranous in these areas suggestive of impending perforation. An additional 18 cm of small bowel was resected (Figure 1).

The diagnosis of RCD II was confirmed upon review by an expert hemato-pathologist of the initial resection specimen, subsequent biopsies, and more recent resection specimens.

**Figure 2:**
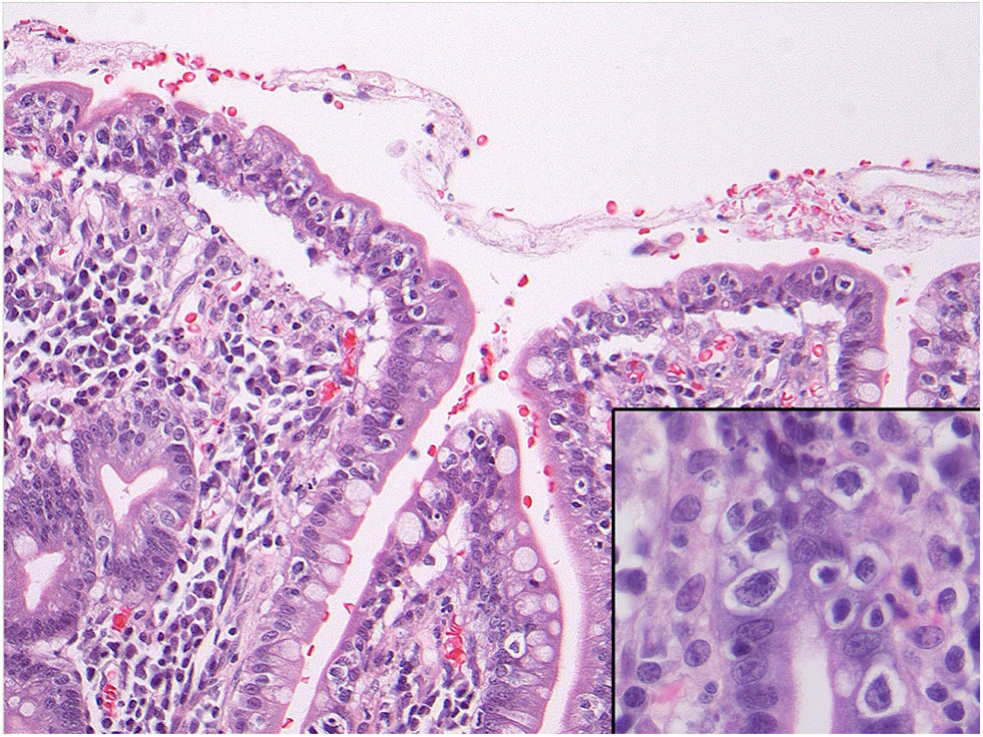
Microscopy of the small intestine showing numerous intraepithelial lymphocytes with perinuclear clearing or halo. The inset shows nuclear atypia of these cells including prominent nucleoli. These cells showed a phenotype compatible with refractory celiac disease type II and also showed monoclonal gene rearrangements.

Abnormal IELs characterized by an abnormal T-cell phenotype and a monoclonal T cell population [as per T-cell receptor gene rearrangement (TCRGR) analysis], consistent with RCD II were identified at various levels of the small and large bowels (with TCRGR studies positive in both). They were accompanied by benign-appearing ulcerations surrounded by a mixed inflammatory population of plasma cells, B-cells and predominant phenotypically normal T lymphocytes and neutrophils, consistent with ulcerative jejunoileitis involving the small bowel and colon. Immunohistochemical assessment confirmed the surface IELs to be CD3(+), positive for CD8 (with major CD8-loss in some of the samples exceeding 80% of IELs), variably positive for TIA-1/granzyme, focally positive for CD30, and entirely negative for CD5 and CD56. EBV *in-situ* hybridization studies were non-contributory. In some of the ulcers, they showed a non-specific slight increase in EBV-positive cells. Most importantly, review of the pathology confirmed that the clones in the intestine and colon were exactly the same (xii). Flow cytometry could not be performed as the tissue from the initial presentation was in formalin (xiii). No evidence of EATL or infection was noted. In addition, the β2 microglobulin and LDH were not elevated, which is typically characteristic of EATL (ii). Furthermore, the biopsies showed no evidence of Inflammatory Bowel Disease and no features of consistent with ischemia (x, xiv) (Figure 2).

He eventually underwent repeat exploratory laparotomy with abdominal washout, lysis of adhesions, gastrostomy tube insertion, proximal small bowel anastomosis, creation of end-loop ileostomy, creation of end colostomy. He was started on IV corticosteroids given the severity of his presentation. Due to his difficult hospital course and prolonged ICU admission and septic complications, IV corticosteroid therapy was held, as the risks outweighed the benefits (iv). He was continued on TPN. His ECOG status was too poor for chemotherapy, and he opted for palliation. He subsequently passed away (v).

## Discussion

Refractory celiac disease (RCD), is defined as persistent or recurrent malabsorption and villous atrophy despite adherence to a strict GFD for 6–12 months or severe persistent symptoms regardless of duration of GFD (3, 4). While patients with RCD I and II differ in presentation and prognosis, they both typically develop resistance to GFD over a period of 3–7 years. On endoscopy, extensive intestinal ulceration (70%) or stenosis is frequently observed in individuals with RCD II, whereas mucosal abnormalities or less common (30%) and milder in patients with RCD I. RCD I usually develops sooner and has a relatively benign course while RCD II is associated with ulcerative jejunitis, lymphocytic gastritis, lymphocytic colitis, and higher rates of enteropathy-associated T- cell lymphoma (EATL) (5–7); hence, establishing the RCD subtype is critical for management and prognosis.

RCD II has a reported incidence of <0.7% in those with confirmed CeD with a mean age of diagnosis of 50 years and a female predominance (8). It classically presents with symptoms of diarrhea, unintentional weight loss, malnutrition, anemia, thromboembolic events, and autoimmune disorders. Hypoalbuminemia is significantly more frequent in RCD II, but not low body mass index nor anemia (5, 8). Similar to EATL, the duration and dose of gluten exposure appear to be risk factors for RCD, as homozygosity for HLA-DQ2 is observed in 44–67% of RCD II and 25–40% of RCD I cases (9, 10) and the majority of RCD patients are older than 50 years. Alleles DQA1^*^05 and DQB1 encode serotypes DQ2, and extended HLA-haplotypes associated with CeD; they are also associated with increased risk of CeD, specifically if an individual carries two copies of DQB1^*^02 (9). HLA-DQ2 homozygosity is more frequent in RCD II and EATL than RCD I (5, 10), as was seen in our patient. Ulcerative jejuno-ileitis is also significantly associated with RCD II, characterized by >1 cm ulcerations in the small bowel; the proximal jejunum is the most common location (5). Transverse circumferential ulcerations have been previously described and can be associated with stricture formation (11) or alternatively described as fissuring type ulceration (12), as seen in this case. Circumferential ulceration can also be seen in EATL (13).

Endoscopically and histologically, lymphocytic gastritis is significantly more common in RCD II, while lymphocytic colitis is seen in up to one-third of patients with both RCD I and II (14–16). Recognition of these features in our case may have facilitated earlier diagnosis. RCD II is also associated with the presence of extraintestinal aberrant IELs, including skin, blood, colon, bone marrow, and liver involvement. Diagnosis confirmation requires demonstration of phenotypically and genotypically aberrant IELs with immunohistochemistry, by flow cytometry and using TCRGR studies (4, 5, 17). RCD I shows increased numbers of phenotypically normal, polyclonal CD8(+) IELs. RCD II is a severe enteropathy, typically presenting with ulcerative jejuno-ileitis and monoclonal or oligoclonal (as per TCRGR studies) expansion of abnormal IELs that are characterized by a lack of expected T-cell surface markers (CD3, CD5, and/or CD8) but preserved expression of intracellular CD3 [hence the common IHC phenotype of CD3+/CD8(–) T-cells]. Flow cytometry with >20% of CD103+ IELs showing an aberrant marker profile is consistent with RCD II. A majority of patients have also been found to have trisomy 1q (4, 8) (Table 1).

**Table 1:** Clinical and Immuno-phenotypic features of Refractory Celiac Disease (RCD) type I and II.

**Features**	**RCD I**	**RCD II**
Female predominance	–	+
Hypoalbuminemia	–	+
Low BMI	±	+
Anemia	+	+
Lymphocytic gastritis	±	+
Lymphocytic colitis	±	±
Extraintestinal Manifestation	–	+
Ulcerative jejunoileitis	–	+
Intra epithelial lymphocytes (IELs)	Normal	Aberrant T-cell IELs Clonal
Surface CD3	+	–
Surface CD8	+	–
Intracellular CD3		+
Trisomy 1q		+

Abdominal imaging may be of additional benefit in RCD II. Non-specific findings, such as bowel-wall thickening or mesenteric lymphadenopathy are seen in both types of RCD; however, cavitating mesenteric lymph node syndrome is suggestive of RCD II or EATL and appears as a cystic change in mesenteric lymph nodes. Small splenic volume (atrophy), intussusception, bowel wall thickening, and lymphadenopathy have also been associated with RCD II and EATL (18). PET has improved sensitivity and specificity in detecting EATL vs. CT abdomen, and should be performed to rule out EATL (18). However, in our case, a PET-CT was not done given the patient severity and limited resources (ix).

Small bowel endoscopy allows for detection and characterization of small bowel lesions, specifically ulcerative jejunitis or large ulcerations (>1 cm) and strictures or ulcerated nodular mucosa, suggestive of malignancy. Video capsule endoscopy (VCE) is not only minimally invasive, but also able to characterize disease extent. However, there is concern for capsule retention if stenosis is present, which is more commonly seen in RCD II; VCE was found to be of little benefit in RCD I. Double-balloon endoscopy (DBE) allows for better detection of suspicious lesions, ulcerative jejunitis, and EATL (19).

Determining a prompt and accurate diagnosis is critical for management of RCD, as the overall 5-year survival in RCD I is 80–90% compared to 44–58% in RCD II (4, 5). The most common cause of death for patients with RCD I is emaciation, secondary to malnutrition; in RCD II, progression to EATL occurs in up to 52% of patients within 5 years following diagnosis vs. 14% in RCD I (4, 5). Risk factors for poor prognosis include age >65 years, hypoalbuminemia (<3.2 g/dL), anemia (Hgb <11 g/dL), presence of aberrant IELs, and total villous atrophy (modified Marsh 3c) at diagnosis (4, 20). Approximately 32–40% of patients with EATL present with small bowel obstruction or perforation, as was the initial presentation in this case. However, while risk factors for poor progression were present, the lymphocytes with abnormal phenotypes were essentially restricted to the surface epithelium, in keeping with RCD type II, and against a diagnosis of EATL. RCD II is considered by many to represent a variant of low-grade lymphoma of intraepithelial T-cell lymphocytes or cryptic EATL.

The current mainstay of treatment of CeD is a GFD, which improves symptoms, corrects nutritional deficiencies, leads to small bowel mucosal recovery, and alters disease progression. As this is a rare entity, there is a paucity of randomized controlled trials studying the efficacy of therapy in RCD, with the majority of data derived from case reports and prospective trials. Corticosteroids have shown clinical benefit with variable histologic improvement, but do not prevent progression to EATL (21). Furthermore, the therapeutic effect is not durable and showed no significant difference when used in combination with thiopurines (22). Given the adverse effects of long-term corticosteroid use, Budesonide has been used as an alternative agent. Retrospective analysis found variable clinical and histologic improvement; however, open-capsule budesonide appeared to be the most clinically effective (5, 23, 24).

Thiopurines also demonstrated variable clinical effects with persistent remnant clonality. Furthermore, they carry an inherent adverse effect of developing lymphoma (6, 8, 25–27). Studies examining methotrexate and cyclosporine failed to differentiate RCD I and RCD II, and thus no meaningful conclusion was drawn (5, 28, 29).

Those with CeD have significantly higher levels of proinflammatory cytokines, including Th-2 cytokines – IL-4 and IL-10 (*P* < 0.001) (30). Mulder et al. (31) studied the use of recombinant human IL-10 in patients that failed corticosteroids, cyclosporine, and azathioprine in a pilot, non-randomized, open label study. It did not achieve its primary endpoint of histologic improvement at 3 months. IFX demonstrated clinical and histologic improvement but did not maintain a durable effect (31–34). Alemtuzumab is an anti-CD52 monoclonal antibody targeting aberrant IELs with CD52 expression typically used to treat chronic lymphocytic leukemia, cutaneous-cell lymphoma, and neurodegenerative disorders. A single case-report showed clinical and histological improvement. Further studies are needed to determine if this is durable and effective therapy (35).

Cladribine (2-CdA) is a synthetic purine nucleoside analog, and its active metabolite, cladribine triphosphate, incorporates itself into lymphocyte DNA, thereby disrupting proliferation, apoptosis, and inflammation (36). It also resulted in both symptomatic improvement and mucosal recovery, as well as decreased aberrant IELs, but did not prevent EATL (5, 6, 36–38). These studies also demonstrate the importance of histologic remission in preventing progression to EATL (39).

More recent developments include Janus kinase inhibitors, blocking IL-15 to decrease IELs, which has shown benefit in transgenic mice models including histologic improvement. CeD disrupts regulation at the cellular level, resulting in overexpression of IL-15 activity and chronic intestinal inflammation, and thus proliferation of IELs. In animal models, Tofacitinib demonstrated histologic improvement, but re-expansion of CD8 T-cells occurred 10 weeks following completion of therapy (3, 13, 40–43) (Table 2).

**Table 2:** Therapeutics and their responses in Refractory Celiac Disease (RCD) type II.

**Therapeutics**	**Clinical symptoms**	**Mucosal recovery**	**Durable response**	**Progression to EATL**
Corticosteroids	Variable	Variable	–	–
Budesonide (Open-capsule)	+	Variable	–	–
Thiopurines	Variable	Persistent remnant clonality	–	–
Infliximab	+	+	–	–
Alemtuzumab	+	+	–	–
Cladribine	+	+		–
JAK Inhibitor	+	+	–^*^	
High-dose chemotherapy with ASCT	+	+	–	–

* *Re-expansion of C8 T-cells, at 10 weeks following completion of therapy in animal models with Tofacitinib*.

Other possible treatments of refractory celiac disease include fecal microbiota and autologous stem cell transplant. In a case report, as with other therapeutic modalities, fecal microbiota demonstrated symptomatic resolution but persistent aberrant IELs. This is the first report to examine the novel concept of the role of the microbiota in CD, and further study is warranted (44). High-dose chemotherapy followed by autologous stem-cell transplant (ASCT) improved biochemical parameters but did not prevent progression to EATL (45–50). Furthermore, the decrease in aberrant IELs was not maintained and there is a paucity of long-term follow-up. Early results suggest ASCT is a safe and effective therapeutic modality for RCD II patients with a high content of aberrant IELs refractory to immunosuppression. The data also supports the use of ASCT in patients who are refractory to Cladribine (45). Despite promising results in RCD II, ASCT has not demonstrated efficacy in EATL, highlighting the importance of identifying therapies that prevent development of EATL (49).

There is a limited role for surgical management aside from complications, such as perforation, obstruction, hemorrhage, or malignancy. It may result in remission if the diseased segment is localized, with improved survival also noted in those with local resection vs. solely treated with chemotherapy (36, 51).

This case is unique in that free perforation is rarely presenting feature of RCDII or lymphoma; this phenomenon has been described in one other case series (2). Moreover, small bowel perforation has been well associated with RCDII and lymphoma; however, colonic perforation is atypical and indicates that any bowel site is at risk for free perforation in CeD associated malignancy. This case highlights the importance of considering CeD and associated complications in the differential diagnosis of free perforation in the intestine (i). We also suspect the vascular insufficiency was multifactorial in etiology, due to hypoperfusion and reperfusion injury. However, thromboembolism in CeD is associated with elevated homocysteine and procoagulant levels (vi). The elevated INR of 9 was likely secondary to Vitamin K deficiency due to malabsorption of fat-soluble vitamins with undiagnosed CeD (vii).

This case presents a common dilemma in the diagnosis of RCD II and highlights the importance of a high index of suspicion, as the diagnosed was significantly delayed in this case (52). Despite a history of hypothyroidism (TSH at admission 20.1) and a family history of CeD, it was felt that the small bowel obstruction was likely due to adhesive disease in the right pelvis, and not thought to be associated with the jejunoileitis secondary to RCDII (iii). However, retrospective reviews of subsequent biopsies and resection samples were consistent with RCD II, both at the phenotypic and genotypic level. In addition, the colonic perforation was suggestive of large bowel involvement, a unique feature of RCD II. Further delaying the diagnosis was the lack of typical symptoms and biochemical abnormalities. The trigger for his severe symptoms at re-presentation is unclear, potentially due to an altered microbiome following surgery in the setting of a normal (gluten-containing) diet. Furthermore, the impact of an earlier diagnosis on the clinical course is unknown, but may have diminished the severity and complications of his disease progression. This case also reiterates the importance of appropriate work-up in nonresponsive CeD, especially in those adhering to GFD, as well as molecular testing to prevent delay in diagnosis, and downstream morbidity and mortality. It also emphasizes the need to better understand the pathophysiology of this entity and guide management as the currently available therapies improve symptoms and histologic features, but do not prevent disease progression.

## Data Availability Statement

The original contributions presented in the study are included in the article, further inquiries can be directed to the corresponding author/s.

## Ethics Statement

Written informed consent was obtained from the individual's next of kin for the publication of any potentially identifiable images or data included in this case report.

## Author Contributions

RC identified the case, conducted the literature search, and prepared the first draft of the manuscript. JN and DM were involved in the surgical aspect and patient care. JD and RM contributed to the pathological part of the study. LD contributed to the gastroenterology interpretation. All authors contributed to the article and approved the submitted version.

## Conflict of Interest

The authors declare that the research was conducted in the absence of any commercial or financial relationships that could be construed as a potential conflict of interest.
